# No difference in stroke knowledge between Korean adherents to traditional and western medicine – the AGE study: an epidemiological study

**DOI:** 10.1186/1471-2458-6-153

**Published:** 2006-06-13

**Authors:** Moon Ho Park, Sangmee Ahn Jo, Inho Jo, Eunkyung Kim, Su-Yong Eun, Changsu Han, Min Kyu Park

**Affiliations:** 1 Department of Biomedical Sciences, National Institute of Health, 5 Nokbun-dong, Eunpyung-gu, Seoul, Korea; 2 The Geriatric Health Clinic and Research Institute (GHCRI), Korea University Medical Center, 516 Gojan-1-dong, Danwon-gu, Ansan-si, Gyeonggi-do, Korea; 3 Department of Psychiatry, Korea University Medical College, 516 Gojan-1-dong, Danwon-gu, Ansan-si, Gyeonggi-do, Korea; 4 Department of Neurology, Korea University Medical College, 516 Gojan-1-dong, Danwon-gu, Ansan-si, Gyeonggi-do, Korea

## Abstract

**Background:**

Effective stroke intervention and risk reduction depend on the general public's awareness and knowledge of stroke. In Korea, where both traditional Oriental medicine and Western medicine are practiced, estimates of the general public's awareness and knowledge of stroke are poor. The present study sought to describe the inception cohort of the Ansan Geriatric Study (AGE study) and to determine baseline stroke awareness and preferred medical treatment for stroke in this Korean sample.

**Methods:**

A total of 2,767 subjects selected randomly from the Ansan Geriatric Study in South Korea were questioned about stroke. Their answers were compared with their sociodemographic data and other variables.

**Results:**

Only 44.8% of participants correctly identified stroke as a vascular disease in the human brain. Sudden numbness or weakness was the most frequently identified stroke warning sign (60.2%). Hypertension (66.7%) and mental stress (62.2%) were most frequently identified as stroke risk factors. The contributions of diabetes mellitus and cardiovascular disease to stroke were underestimated; they were identified as risk factors by 28.3% and 18.6% of participants, respectively. The predictors for poor knowledge of stroke warning signs and risk factors were similar irrespective of preference for Western or Oriental medical treatment, and included those with lower levels of education and inaccurate definition of stroke. Television and radio (40.3%) were the most frequent sources of stroke information for both groups.

**Conclusion:**

This study shows that knowledge of stroke is similar among Koreans with preferences for either Western or Oriental medical treatment and that misunderstandings about stroke are common among the Korean elderly. In order to prevent and manage stroke effectively, public health education regarding basic concepts of stroke is necessary. This should target those with a lower level of education and a misunderstanding of the definition of stroke.

## Background

Stroke is the leading cause of death among Koreans [1,2]. Control of stroke risk factors and, when stroke occurs, early initiation of treatment, represent primary steps in stroke prevention and reduction of mortality, sequelae and length of hospitalization [3,4]. However, inadequate or incorrect knowledge about stroke among the general public may hinder prevention and delay the initiation of appropriate treatment. Thus, it is important to increase public awareness of stroke signs and symptoms and potential stroke risk factors [5,6]. Accurate estimation of public awareness and knowledge of stroke is necessary for improving understanding among the general public.

While traditional Oriental medicine is considered 'alternative medicine' by most of the world, it is as respected as Western medicine in South Korea [7,8] and for a long time has played an important role in disease treatment. However, few epidemiological reports have addressed differences in knowledge about stroke between those who prefer Western and those who prefer traditional Oriental medicine.

With the overarching goal of enhancing the success of primary and secondary stroke care strategies in Korea, the present study sought to describe the inception cohort of the Ansan Geriatric Study (AGE study) and to determine baseline stroke awareness and preferred medical treatment for stroke in this Korean sample.

## Methods

### Study design and population

The AGE study was initiated in May 2002 to perform population-based cohort research on the Korean elderly population, as Korean society is now considered to be one of the most rapidly aging [2]. The overall objective of the AGE study is to provide not only basic information on the medical condition and characteristics of the rapidly growing Korean geriatric population, but also to identify genetic and environmental risk factors for various diseases. As part of a baseline investigation to construct an elderly cohort in Korea, we sampled the elderly population living in Ansan, located in the southern part of Gyeonggi-do province, South Korea. Ansan comprises rural and urban areas and is now included as a metropolitan area of Seoul. In August 2002, 36,735 non-institutionalized civilians aged 60 to 84 years were registered as residing in Ansan [2]. On the basis of a power analysis of the sample size needed to determine the prevalence of major chronic diseases and risk factors among the elderly, a goal of interviewing 3,000 subjects was set.

To construct the sampling framework, telephone-subscriber data from the Korean Telecommunications Corporation, a near-monopolist telephone company in Korea, were compared with residence records. Among the 36,735 non-institutionalized civilians, a total of 19,387 records (52.8%) were matched to telephone number using name and address as the key variables; the remaining 17,348 residents (47.2%) did not have matched telephone numbers. To acquire a probability sample proportional to the age- and gender-specific population structure of the target population, a random sample comprising 15,392 persons (41.9%) aged 60 to 84 years was selected: 10,819 individuals from the group with matched telephone numbers, and 4,573 from the unmatched group. A letter of invitation to participate in this study was initially sent to all 15,392 individuals. The telephone-number-matched group was additionally contacted at least three times by telephone, and the unmatched group was visited at home in order to confirm their eligibility. A total of 8,999 individuals were determined ineligible for interview for 'logistical and unknown' reasons such as incorrect telephone number, incorrect address or change of address, moving, death, and absence at three attempted visits. Another 1,989 individuals refused our study. This left 4,404 eligible individuals (12% of the target population or 28.6% of the stratified random sample) for in-home or in-clinic interview. After excluding persons who refused, provided incomplete data or were ineligible for a probability sample proportional to the age- and gender-specific structure of the target population, the final sample comprised 2,767 elderly Koreans representative of the target population.

The study was approved by the Institutional Review Boards of the Korea University Medical Center, Ansan Hospital. Written informed consent was obtained from all participants or their relatives after the study's procedures were fully explained.

### Procedures

Participants were asked to choose between two interview locations, their home or the Geriatric Health Clinic and Research Institute (GHCRI) of the Korea University Hospital, according to their convenience. Approximately half the study participants (46.3%) were interviewed in their homes, while 53.7% were interviewed at the clinic. A survey team composed of trained field surveyors was instructed in the details of the questionnaires and measurements before the survey was initiated. A team of two surveyors interviewed the participants to collect all data. Each study participant was interviewed by one surveyor, while a second surveyor asked another member of the household to confirm the sociodemographic data. To avoid bias the survey team identified sociodemographic data only after the completion of the interview. McNemar's test was used to assess surveyor reliability and revealed no significant difference (p > 0.05).

A total of 1,552 participants (56.1%) were women and 1,215 (43.9%) were men. The mean (SD) age of the participants was 68.39 (6.06) years (range 60–84 years). The mean (SD) educational duration of the participants was 6.97 (4.96) years (range 0–25 years). The distribution of household income among the participants was as follows: 871 participants (31.5%), < 500,000 Korean won; 706 (25.5%), 500,000–999,999 Korean won; 1169 (42.2%), > 1,000,000 Korean won (1 U.S. dollar = 1,055 Korean won at the time of the interviews). A total of 1,824 participants (65.9%) were married or cohabitating, and 943 (34.1%) were single (unmarried, separated, divorced or widowed).

The survey team systematically interviewed the subjects regarding their knowledge and perceptions of stroke and stroke treatment, including their understanding of how stroke was defined, their preferred medical treatment in the case of stroke, their knowledge of stroke warning signs and risk factors, and their sources of information about stroke. To determine respondents' knowledge of important stroke warning signs, respondents were asked the following open-ended question: "Can you tell me what the warning signs or symptoms of stroke are?" The responses were coded into five pre-existing categories based on those used by several national organizations (Korean Stroke Society [9], American Stroke Association [10] and National Institute of Neurological Disorders and Stroke [11]), which in their educational materials list the following as important warning signs of stroke: (1) sudden numbness or weakness of the face, arm or leg, especially on one side of the body; (2) sudden confusion or difficulty in speaking or understanding speech; (3) sudden visual impairment in one or both eyes; (4) sudden difficulty in walking, dizziness, or loss of balance or coordination; and (5) sudden severe headache with no known cause. Interviewers were provided with several alternative wordings for the warning signs. Responses considered incorrect included shortness of breath, pain in the chest or arm, and tremor. Loss of consciousness, neck stiffness and seizure were classified as neither correct nor incorrect responses.

Knowledge of risk factors was assessed by identifying the following established risk factors as correct responses [12]: hypertension, smoking, hyperlipidemia, cardiovascular disease, age, diabetes, race, gender, physical inactivity, poor diet, obesity and heredity. Stress, overexertion, low financial status, liver disease, lung disease, kidney disease and gastrointestinal disease were regarded as incorrect responses. Alcohol consumption was classified as neither correct nor incorrect.

Participants' preferred medical treatment for stroke was assessed using the following question: "If you have a stroke, where would you go for treatment?" The response was coded as either traditional Oriental or Western medicine.

### Statistical analysis

Univariate analyses were used to investigate the relationship between each answer in the questionnaire and age, gender, marital status, level of education, financial status, family history of stroke, definition of stroke, and preference for Oriental versus Western stroke treatment. Odds ratios (OD) and 95% confidence intervals (CI) were generated for all outcomes of interest. Factors determined to be potentially associated with stroke awareness were entered simultaneously into a logistic regression model to assess independence. A previous study by Schneider and colleagues [13] using serial surveys showed a statistically significant difference in knowledge of at least two stroke risk factors, and Silver and colleagues [14] used the ability to name at least two stroke warning signs as the main outcome measure. Thus, to enable comparisons to previous studies to be made, the ability to recognize ≥2 stroke risk factors and stroke warning signs was used as the main outcome measure in this study. Logistic regression was used to determine the significance of various sociodemographic characteristics and other variables as predictors of knowledge of at least two stroke risk factors and stroke warning signs. Logistic regression models were developed using Enter methods. In all analyses, a probability value of p < 0.05 was considered statistically significant.

## Results

Stroke was most frequently defined as a cerebrovascular disease (n = 1,214, 44.8%); other definitions (in decreasing order of frequency) were an aging process in the brain (n = 179, 6.6%), peripheral nervous disease (n = 70, 2.5%) and convulsive disorder (n = 53, 1.9%). A total of 779 subjects (28.2%) answered that they did not know.

When asked where they would seek treatment for a stroke, 1,625 of the subjects (58.7%) indicated that they would select Western medical treatment and 1,142 (41.3%) indicated that they would select traditional Oriental medicine. The most frequent reason for selecting Western medicine was that it is more scientific than traditional Oriental medicine (n = 656, 41.7%). The respondents who selected traditional Oriental medicine answered that traditional Oriental medicine has a greater curative effect (n = 462, 53.2%), and this is a general trend that means many other people select traditional Oriental medicine (n = 150, 17.3%).

The stroke warning sign most frequently identified by respondents was sudden numbness or weakness (n = 1,667, 60.2%), followed by sudden confusion or difficulty speaking or understanding speech (n = 607, 21.9%). There was no significant difference in perception of stroke warning signs between groups preferring traditional Oriental and Western medicine (Table 1). Only 623 respondents (24.3%) correctly listed at least two important warning signs of stroke. In the univariate comparison, older age (p = 0.013), lower level of education (p < 0.001) and incorrect knowledge of the definition of stroke (p < 0.001) were predictive of poorer knowledge of the important stroke warning signs (Table 2).

Respondents' answers relating to the most important risk factors for stroke are summarized in Table 3. Hypertension (n = 1,846, 66.7%), stress (n = 1,720, 62.2%), overexertion (n = 1,325, 47.9%), obesity (n = 1,270, 45.9%) and smoking (n = 1,216, 43.9%) were the five most common answers. As was the case with stroke warning signs, there was no significant difference in perception of stroke risk factors between groups that favored treatment with traditional Oriental versus Western medicine. A total of 1,753 respondents (68.3%) correctly identified at least two established stroke risk factors. In univariate comparison, knowledge of stroke risk factors was associated with age (p < 0.001), gender (p < 0.001), marital status (p = 0.004), level of education (p < 0.001), financial status (p < 0.001), family history of stroke (p < 0.001), knowledge of the definition of stroke (p = 0.001) and preferred medical treatment for stroke (p = 0.004) (Table 4).

In the multivariate logistic regression model, a lower level of education and incorrect knowledge of the definition of stroke were associated with poorer knowledge of important stroke warning signs. Older age, lower level of education and incorrect knowledge of the definition of stroke were significant predictors of poorer knowledge of stroke risk factors (Table 5).

The most popular sources of information about stroke were certain components of the mass media (i.e. television and radio, n = 1,091, 40.3%). Other sources of information (in order of decreasing frequency) were family member or other relative (n = 387, 14.3%), newspapers or magazines (n = 173, 6.4%), medical institute (n = 151, 5.6%), school (n = 5, 0.2%) and the Internet (n = 3, 0.1%). Those participants who preferred Western medicine for stroke treatment obtained their information and knowledge from television or radio (n = 718, 47.5%), a family member or other relative (n = 194, 7.9%) and newspapers or magazines (n = 138, 9.1%). Participants who preferred Oriental medicine for stroke treatment derived most of their information about stroke from television or radio (n = 373, 35.3%) and a family member or other relative (n = 193, 18.2%) (χ^2 ^= 126.035, degrees of freedom = 7, p = 0.001).

## Discussion

Evaluating general public awareness can help to improve public information programs about stroke, but our estimates indicate that stroke awareness among Koreans is generally poor. Only 44.8% of the subjects in this study answered correctly that stroke is a vascular disease in the brain. The inability of many Koreans to define stroke correctly may be attributable to the strong influence of traditional Oriental medicine, which defines stroke as 'paralysis by lack of harmony of negativity and positivity' and considers stroke a disease of both the peripheral nervous and cerebrovascular systems [15]. Traditional Oriental and Western medicine have equal social and cultural status in the treatment of stroke in Korea; indeed, the Chinese character 'Poong' (meaning wind) has spread to the Western medical terminology for stroke [7,8]. However, we found that preference for Oriental versus Western medicine for stroke treatment did not significantly affect awareness of stroke warning signs and risk factors. The lack of difference may be attributable to Korea's modernization and industrialization, which have led to the dissipation of traditional concepts and the emergence of Western medicine campaigns by groups such as the Korean Stroke Society [9].

Public knowledge of stroke warning signs is important for ensuring timely access to emergency medical care [14,16,17]. Our results show numbness and weakness to be the most frequently (and correctly) identified signs and symptoms of stroke (60.2%). A higher proportion of respondents recognized numbness and weakness as a stroke warning sign. In four previous studies, the most common stroke warning signs listed by respondents were dizziness (24%) and numbness (19%) [5], weakness (26%) [18], blurred vision (24%) [19], and numbness (45%) and speech difficulties (38%) [20]. Differences in the medical systems and location of the sample populations make it difficult to compare the present study with other population studies. However, it is possible that the variation in stroke warning signs identified by the study participants reflects differences in their perceptions of the importance or severity of symptoms. It was encouraging that more than 60% of respondents in this study reported the correct signs of stroke.

Stroke prevention is dependent on the ability to recognize and control stroke risk factors [5,6,21]. Increased public awareness of potential risk factors will help to reduce the prevalence of stroke and increase treatment compliance. The proportion of respondents considering hypertension to be a risk factor of stroke was higher in our study (66.7%) than in a previous study in Korea (28.3%) [8]. The better understanding of potential hypertension risks in our study may be attributable to the special campaigns conducted by the National Hypertension Center of the Korean government over the past few years [22,23]. As in previous studies, diabetes mellitus and cardiovascular disease were underestimated as risk factors, while mental stress and overexertion were considered important [5,8,18]. Given the lack of evidence that mental stress and overexertion are stroke risk factors, it is important to emphasize the contributions of diabetes mellitus, cardiovascular disease, obesity and smoking to stroke [24]. In this study, answers to the questionnaire differed significantly depending on whether or not the respondent was able to define stroke correctly. This suggests that understanding of the basic concepts of stroke may play a crucial role in public health.

In the multivariate logistic regression model, a lower level of education and incorrect definition of stroke were independent predictors of poorer knowledge of stroke warning signs. These finding are consistent with those of a previous study [20] reporting that younger age, female gender, higher level of education and high cholesterol were all associated with the correct identification of stroke warning signs. We also found that older age, lower level of education, family history of stroke and incorrect definition of stroke were independent predictors of poorer knowledge of stroke risk factors. Blades and colleagues [20] also showed that younger respondents and women were more likely to identify stroke risk factors correctly. The overall implication of the present study's findings is that these populations should be specifically targeted with public health education about stroke.

Despite the tendency among Koreans to respect and follow the decisions of the oldest member of their families (who tend to prefer traditional Oriental medicine [8]), Western medicine was preferred over traditional Oriental medicine for medical treatment of stroke. The customary idea in Korea that stroke should be treated by traditional Oriental medicine may lead to the expectation that traditional Oriental medicine produces better results. However, the actual effectiveness of traditional Oriental medicine for stroke is controversial [25]. One of the main reasons that patients are not admitted to a hospital early enough in Korea is that they seek treatment with traditional Oriental medicine beforehand [8].

The mass media (television, radio, newspapers and magazines) were the most popular sources of information for stroke knowledge; this should be considered when promoting public knowledge of stroke and the associated risk factors [13]. In contrast, our findings suggest that hospitals and medical schools do not provide effective public education. Public education promoting awareness of the seriousness of stroke and provision of access to emergency care during the narrow therapeutic time window may lead to changes in behavior [13]. A previous study evaluating the effectiveness of different media in increasing public awareness of stroke found that television advertising is often promoted owing to its ability to reach across demographic groups based on gender, age and education [14]. The second most popular source of information about stroke was a family member or other relative, suggesting that acquisition of information from personal contacts could become an effective method for dispersing accurate medical information. Ensuring that patients and their families retain simple and accurate information on stroke as they progress through stroke rehabilitation may be an effective means of disseminating this information to the general public [18].

This study has a number of limitations. The study was conducted on a Korean population, and there may be significant differences with respect to the awareness of stroke warning signs and risk factors in other geographic and racial and ethnic communities. Moreover, because the sample was drawn from a limited geographic area within Korea, the results may not generalize to the national population. However, many rural districts in Korea are quickly urbanizing, and Ansan is a good example of the nation's many urbanized areas. The relatively low sample size may also affect the study's generalizability, although the sampling design does represent a random age- and gender-stratification. There is also the possibility of non-coverage and non-response bias because those not sampled in this study may have a different level of stroke awareness than those who participated. Aside from generalizability, another limitation of the study is that it does not address whether there is a causal relationship between stroke knowledge and behavior such as risk factor modification and rapid seeking of medical attention in the event of stroke symptoms.

## Conclusion

Our study has confirmed the need for more public education about stroke, targeted in particular at those who are less educated, currently unaware of the correct definition of stroke, or elderly, regardless of whether they prefer Western or traditional Oriental medicine. This education should include information about risk factors, warning symptoms and the methods used to prevent and manage stroke.

## Competing interests

The author(s) declare that they have no competing interests.

## Authors' contributions

Park MH and Jo SA: principle investigators of the study. They were involved in the study planning, discussion of the project, and manuscript writing. Jo I: main research assistant of study. He managed and cleaned the data set, analyzed the data for this study. Kim E: main data-manager. She was involved in the study planning and was responsible for data collection and data analysis. Eun SY: internal statistician. She was involved in study planning, definition of outcome, statistical analysis, and draft writing. Han C: lead investigator. He was involved in coordinating the 2003 GHCRI study and contributed to the intellectual discussion of the concept of the article. Park MK: principal investigator. He was involved in coordinating the 2002 GHCRI study and was contributed in the intellectual discussion of the concept of the article and the idea of data analysis. All authors read and approved the final manuscript.

## Pre-publication history

The pre-publication history for this paper can be accessed here:



## Figures and Tables

**Table 1:** Respondents' reported stroke warning signs

Warning sign	Preferred medical treatment*	
	Western	Oriental Traditional
*Correct responses*		
Sudden numbness or weakness	926 (57.0%)	741 (64.9%)
Sudden confusion, or difficulty speaking, or understanding speech	361 (22.0%)	246 (21.5%)
Sudden difficulty walking, dizziness or loss of coordination	258 (15.9%)	136 (11.9%)
Sudden severe headache	66 (4.1%)	27 (2.4%)
Sudden visual impairment	10 (0.6%)	7 (0.6%)
		
*Incorrect responses*		
Tremor	108 (6.6%)	69 (6.0%)
I don't know	169 (10.4%)	120 (10.5%)
Other miscellaneous^†^	194 (11.9%)	110 (9.6%)
		
*Neither*		
Loss of consciousness	81 (5.0%)	56 (4.9%)
Other miscellaneous^‡^	6 (0.4%)	6 (0.4%)

*P > 0.05

^†^shortness of breath, pain in chest or arm, seizure, and sweating

^‡^neck stiffness, and quadriplegia

**Table 2:** Univariate logistic regression: factors related to knowledge of at least two stroke warning signs

	Number (%)	Correct knowledge %	OR (95%CI)	*P*
Age, years				
60–64	910 (32.9)	25.4	*reference*	0.013
65–69	775 (28.0)	25.5	1.01 (0.81–1.26)	
70–74	554 (20.0)	24.2	0.94 (0.76–1.24)	
75–79	348 (12.6)	19.5	0.71 (0.53–0.97)	
80–84	180 (6.5)	15.6	0.54 (0.35–0.83)	
				
Gender				
Male	1215 (43.9)	25.3	*reference*	>0.05
Female	1552 (56.1)	22.6	0.86 (0.72–1.03)	
				
Marital status				
Married/cohabiting	1842 (65.9)	24.2	*reference*	>0.05
Single*	943 (34.1)	23.1	0.94 (0.78–1.14)	
				
Education level				
High	303 (11.0)	30.7	*reference*	<0.001
medium	939 (33.9)	27.4	0.85 (0.64–1.13)	
Low	1025 (37.0)	22.5	0.66 (0.49–0.87)	
Illiterate	500 (18.1)	15.6	0.42 (0.29–0.59)	
				
Household income				
High	1169 (42.6)	25.1	*reference*	>0.05
Medium	706 (25.7)	21.8	0.83 (0.67–1.04)	
Low	871 (31.7)	23.0	0.89 (0.72–1.09)	
				
Family history of stroke				
Family history	470 (17.0)	77.2	*reference*	>0.05
No family history	2297 (83.0)	64.3	0.84 (0.67–1.05)	
				
Knowledge of the definition of stroke				
Correct	1214 (43.9)	28.7	*reference*	<0.001
Incorrect	1553 (56.1)	20.0	0.62 (0.52–0.74)	
				
Preferred medical treatment				
Western	1625 (58.7)	24.7	*reference*	>0.05
Oriental Traditional	1142 (41.3)	22.5	1.13 (0.95–1.35)	

*unmarried, separated, divorced, or widowed

**Table 3:** Respondents' reported stroke risk factors

Risk factor	Preferred medical treatment*	
	Western	Traditional Oriental
*Correct responses*		
Hypertension	1135 (69.8%)	711 (62.3%)
Obesity	787 (48.4%)	483 (42.3%)
Smoking	758 (46.6%)	458 (40.1%)
Hyperlipidemia	670 (41.2%)	419 (36.7%)
Age	696 (42.8%)	355 (31.1%)
Heredity	563 (34.6%)	368 (32.2%)
Diabetes	515 (31.7%)	268 (23.5%)
Cardiovascular disease	355 (21.8%)	159 (13.9%)
		
*Incorrect responses*		
Stress	1039 (63.9%)	681 (59.6%)
Overexertion	804 (49.5%)	521 (45.6%)
Financial status	283 (17.4%)	162 (14.2%)
Liver disease	147 (9.0%)	85 (7.4%)
Pulmonary disease	95 (5.8%)	64 (5.6%)
Kidney disease	85 (5.2%)	53 (4.6%)
Other^†^	25 (1.5%)	27 (2.4%)
I don't know	115 (7.1%)	164 (14.4%)
		
*Neither*		
Alcohol	805 (49.5%)	504 (44.1%)

*P > 0.05

^†^depression, destiny, and gastrointestinal disease

**Table 4:** Univariate logistic regression: factors related to knowledge of at least two stroke risk factors

	Number (%)	Correct knowledge %	OR (95%CI)	*P*
Age, years				
60–64	910 (32.9)	72.7	*reference*	<0.001
65–69	775 (28.0)	70.7	0.93 (0.75–1.15)	
70–74	554 (20.0)	64.6	0.70 (0.56–0.88)	
75–79	348 (12.6)	56.3	0.50 (0.38–0.64)	
80–84	180 (6.5)	44.4	0.31 (0.22–0.43)	
				
Gender				
Male	1215 (43.9)	70.2	*reference*	<0.001
Female	1552 (56.1)	63.5	0.74 (0.63–0.87)	
				
Marital status				
Married/cohabiting	1842 (65.9)	68.3	*reference*	0.004
Single*	943 (34.1)	62.9	0.79 (0.67–0.93)	
				
Education level				
High	303 (11.0)	76.2	*reference*	<0.001
Medium	939 (33.9)	72.2	0.81 (0.60–1.09)	
Low	1025 (37.0)	65.6	0.59 (0.44–0.80)	
Illiteracy	500 (18.1)	51.6	0.33 (0.24–0.46)	
				
Household income				
High	1169 (42.6)	69.0	*reference*	0.001
Medium	706 (25.7)	68.8	0.99 (0.81–1.21)	
Low	871 (31.7)	61.4	0.71 (0.59–0.86)	
				
Family history of stroke				
Family history	470 (17.0)	77.2	*reference*	<0.001
No family history	2297 (83.0)	64.3	0.53 (0.42–0.67)	
				
Knowledge of the definition of stroke				
Correct	1214 (43.9)	75.4	*reference*	0.001
Incorrect	1553 (56.1)	59.5	0.48 (0.40–0.57)	
				
Preferred medical treatment				
Western	1625 (58.7)	68.6	*reference*	0.004
Oriental Traditional	1142 (41.3)	63.4	0.79 (0.68–0.93)	

*unmarried, separated, divorced, or widowed

**Table 5:** Multivariate logistic regression: factors related to knowledge of at least two stroke warning signs or risk factors

Factor	OR (95% CI)
**Knowledge of at least two stroke warning signs**	
Age (years)	
65–69	1.05 (0.84–1.31)
70–74	1.02 (0.79–1.30)
75–79	0.83 (0.61–1.14)
80–84	0.67 (0.43–1.04)
Education level	
Medium	0.87 (0.65–1.15)
Low	0.66 (0.48–0.91)
Illiteracy	0.46 (0.31–0.68)
Incorrect knowledge of stroke definition	0.71 (0.59–0.85)
	
**Knowledge of at least two stroke risk factors**	
Age (years)	
65–69	0.98 (0.77–1.22)
70–74	0.78 (0.62–0.99)
75–79	0.62 (0.47–0.81)
80–84	0.39 (0.27–0.55)
Gender	
Female	0.95 (0.79–1.14)
Education level	
Medium	0.86 (0.63–1.17)
Low	0.72 (0.52–0.99)
Illiteracy	0.50 (0.35–0.71)
Household income	
Medium	0.99 (0.80–1.22)
Low	0.85 (0.70–1.03)
Family history of Stroke	0.59 (0.47–0.75)
Incorrect knowledge of stroke definition	0.59 (0.49–0.70)
Preferred medical treatment to traditional Oriental medicine	0.96 (0.80–1.14)
